# Impact of airway challenges on cardiovascular risk in asthma – a randomized controlled trial

**DOI:** 10.1371/journal.pone.0288623

**Published:** 2023-07-17

**Authors:** Linn E. Moore, Andrew R. Brotto, Desi P. Fuhr, Rhonda J. Rosychuk, Eric Wong, Mohit Bhutani, Michael K. Stickland

**Affiliations:** 1 Pulmonary Division, Department of Medicine, Faculty of Medicine and Dentistry, University of Alberta, Edmonton, Alberta, Canada; 2 Faculty of Kinesiology, Sport, and Recreation, University of Alberta, Edmonton, Alberta, Canada; 3 Department of Pediatrics, Faculty of Medicine and Dentistry, University of Alberta, Edmonton, Alberta, Canada; Srebrnjak Children’s Hospital, CROATIA

## Abstract

**Background:**

People experiencing asthma exacerbations are at increased risk of cardiovascular events. To better understand the relationship between asthma exacerbations and cardiovascular risk, this randomized case-control, cross-over controlled trial assessed the immediate systemic inflammatory and vascular responses to acutely induced pulmonary inflammation and bronchoconstriction in people with asthma and controls.

**Methods:**

Twenty-six people with asthma and 25 controls underwent three airway challenges (placebo, mannitol, and methacholine) in random order. Markers of cardiovascular risk, including serum C-reactive protein, interleukin-6, and tumor necrosis factor, endothelial function (flow-mediated dilation), microvascular function (blood-flow following reactive hyperemia), and arterial stiffness (pulse wave velocity) were evaluated at baseline and within one hour following each challenge. The systemic responses in a) asthma/control and b) positive airway challenges were analyzed. (ClinicalTrials.gov reg# NCT02630511)

**Results:**

Both the mannitol and methacholine challenges resulted in clinically significant reductions in forced expiratory volume in 1 second (FEV_1_) in asthma (-7.6% and -17.9%, respectively). Following positive challenges, reduction in FEV_1_ was -27.6% for methacholine and -14.2% for mannitol. No meaningful differences in predictors of cardiovascular risk were observed between airway challenges regardless of bronchoconstrictor response.

**Conclusion:**

Neither acutely induced bronchoconstriction nor pulmonary inflammation and bronchoconstriction resulted in meaningful changes in systemic inflammatory or vascular function. These findings question whether the increased cardiovascular risk associated with asthma exacerbations is secondary to acute bronchoconstriction or inflammation, and suggest that other factors need to be further evaluated such as the cardiovascular impacts of short-acting inhaled beta-agonists.

## Introduction

Asthma is a chronic airway disease characterized by recurrent episodes of pulmonary inflammation leading to bronchoconstriction and symptoms such as breathlessness, wheezing, and chest tightness [1]. People with asthma have an increased risk of developing cardiovascular disease [2, 3], and the cardiovascular disease risk tends to increase with worsening asthma severity [4–8]. A recent report showed that people experiencing asthma exacerbations requiring hospital treatment were five times more likely to suffer a first myocardial event the week following the asthma exacerbation compared to the months immediately before the episode [9]. While the reasons for the increased cardiovascular disease risk seen during asthma exacerbations are largely unknown, there are different aspects of less well-controlled asthma which may contribute to cardiovascular risk, including elevated inflammation [10–13] and vascular dysfunction [5, 6].

Findings from animal studies suggest that increased levels of systemic inflammation during asthma-like events originate in the lungs [14, 15]. Indeed, markers of acute inflammation, such as C-reactive protein (CRP), are elevated in exhaled breath condensate during naturally occurring asthma exacerbations, and are correlated with CRP levels measured systematically at the same time-points [8, 10]. Elevated systemic inflammation has also been shown to acutely compromise cardiovascular health in otherwise healthy individuals [16, 17]. It is still unknown if the increased cardiovascular risk seen acutely following asthma exacerbations is a consequence of the pulmonary inflammatory insult leading to systemic inflammation and vascular impairment.

The mannitol airway challenge is a widely accepted to identify airway hypersensitivity. Inhaled mannitol is known to activate mast cells within the airways [18, 19], triggering the release of inflammatory mediators and cytokines similar to what is observed during a naturally occurring asthma exacerbation [20]. Therefore, the mannitol challenge leads to bronchoconstriction secondary to airway inflammation in susceptible individuals [18, 19, 21, 22]. Inhaled methacholine binds to muscarinic receptors on the airway smooth muscles, and in susceptible individuals causes a reduction in lung function independent of airway inflammation [23, 24]. Evaluating the responses to mannitol and methacholine challenges separately would allow us to evaluate the systemic inflammatory and cardiovascular responses to bronchoconstriction alone (i.e. methacholine) versus airway inflammation and bronchoconstriction (i.e mannitol). As such, the purpose of this study was to utilize both mannitol and methacholine airway challenges to assess the impact of airway inflammation and bronchoconstriction on systemic inflammation and vascular function as markers of cardiovascular disease risk in people with and without asthma in a controlled laboratory environment. We hypothesised that the mannitol challenge would lead to a decline in vascular function compared to a placebo challenge and that the systemic responses to the bronchial challenges would be potentiated among people with confirmed asthma compared to healthy controls. As a positive mannitol challenge may be due to a greater inflammatory response, we further explored the hypothesis that there would be different vascular and inflammatory response among those with positive responses to mannitol than methacholine or placebo.

## Methods

This case-control, cross-over randomized controlled clinical trial-study was approved by the University of Alberta Ethics Board (Pro0054047), Health Canada (#9427-G0890-88C), registered on ClinicalTrials.gov (NCT02630511), and conducted according to CONsolidated Standards of Reporting Trials (CONSORT) guidelines. All participants signed written informed consent in the presence of a witness prior to participating in the study. The researchers had access to information that could identify participants during and after data collection. There were no adverse events reported.

### Research participants

Patients with physician-diagnosed asthma between the ages of 18 and 45 years were identified by chart review from the University of Alberta Asthma Clinic and The Lung Health Clinic, Edmonton, Alberta 2015–2021. Asthma was confirmed if the participant tested positive for one of the following: a) more than 12% and 200 ml reversibility in the forced expiratory volume in 1 second (FEV_1_) with salbutamol [23], b) a reduction in FEV_1_ of more than 20% at a provocative concentration (PC_20_) of methacholine of less than 4 mg/ml [23], or c) 10% reduction in FEV_1_ following exercise [23]. Those with known lung conditions other than asthma, known cardiovascular disease, and a body mass index (BMI) ≥35 kg/m^2^ were excluded from the study. Control participants fulfilling the same criteria but without a clinical history of asthma and with negative results on all asthma screening tests were recruited from the general population.

### Study design

Following signing informed consent, reporting medical history, and filling out the Asthma Control Questionnaire (ACQ) [25], each subject performed a full pulmonary function test [26–28] and a cardiopulmonary exercise test. These data were used to characterize study participants and to identify potential unknown underlying CV disease for exclusion from the study.

The subsequent study consisted of three experimental days where the participant received either: 1) mannitol airway challenge, 2) methacholine airway challenge, or 3) saline airway challenge (i.e. placebo). The order of challenges was computer-randomized independently from allocation and the subjects were blinded to the type of airway challenge administered. Because of potential bronchoconstriction, it was not possible to fully blind the researchers obtaining post-challenge data. However, vascular and inflammatory data were analyzed blind to the intervention. Each visit occurred at a minimum of one week apart to allow for recovery and to minimize potential carry-over effects between challenges. All participants were asked to withhold caffeinated drinks, food, alcohol, and exercise for a minimum of 12 hours prior to each study visit. While no changes were made to individual medication plans, all participants withheld short-acting beta-agonists for eight hours and long-acting controller medication for 48 hours prior to each test day [29]. All tests were done at the same time of day in the morning to minimize circadian influences on testing outcomes.

Each test visit started with the participant resting in the supine position for 10 minutes in a dimly lit room. Baseline brachial blood pressure was measured in duplicate, and a stable baseline was established when the variance between systolic blood pressure measurements was less than five percent. Arterial stiffness and vascular function were then evaluated. Serum was collected for systemic inflammatory measurements. Following baseline measurements, participants completed one of the three bronchial challenges. For consistency, independent of the airway response to the given intervention, each participant received 400°g salbutamol inhalation powder at the end of each intervention, within five minutes of challenge termination. Follow-up testing occurred within one hour after each bronchial challenge, and all measurements were repeated in the same order as at baseline.

#### Mannitol

The mannitol challenge was performed according to manufacturer guidelines [30]. Each participant received incremental doses of mannitol (0, 5, 10, 20, 40, 80, 160, 160, and 160mg) every two minutes up to a maximal cumulative dose of 635 mg. Briefly, following baseline spirometry measurements, the participant was instructed to fully inhale the content of the inhaler provided from the manufacturer containing the mannitol capsule (Aridol®, Pharmaxis, Frenchs Forest, NSW, Australia). The participant then held their breath for five seconds, exhaled fully, then performed two forced vital capacity maneuvers one minute later. The test ended when either a) the FEV_1_ was reduced more than 10 percent of baseline values (indicating a positive test), or b) completion of the last 160mg mannitol concentration.

#### Methacholine

The methacholine challenge was performed using the incremental two-minute tidal breath-protocol [29]. Following baseline spirometry, the participant was instructed to breathe through a facemask connected to a nebulizer (Wright, Roxon Medi-Tech, Montreal, QC, Canada) with saline (baseline) for two minutes. Following saline, a small amount of methacholine was added to the nebulizer (0.03 mg/ml), and the participant breathed through the facemask for another two minutes. For subsequent stages, the concentration of methacholine was doubled for each round of inhalations (0.031, 0.062, 0.125, 0.25, 0.5, 1.0, 2.0, 4.0, 8.0, 16.0 mg/ml). Spirometry was performed at 30 seconds and 90 seconds following each concentration. The test ended when either a) FEV_1_ was reduced more than 20 percent of baseline values (indicating a positive test), or b) completion of the 16 mg/ml methacholine concentration.

#### Placebo

The placebo challenge was performed identical to the methacholine challenge; however, no methacholine was added to the inhaled saline (i.e. participant inhaled saline only). The challenge was terminated following five rounds of saline.

### Outcome measurements

#### Systemic inflammation

Ten ml of blood was collected from a vein in the antecubital fossa using standard venipuncture technique. The samples were allowed to coagulate for a minimum of 30 minutes at room temperature, then centrifuged at 12,000 rpm for 10 minutes. Serum was then collected and aliquoted into samples of 100°L and stored in a -80 degrees Celsius freezer. Analyses of C-reactive protein (CRP), interleukin-6 (IL-6), and tumor necrosis factor (TNF) levels was done in duplicates using immunofluorescent technique (CRP DuoSet ELISA kit; Human IL-6 Quantikine ELISA Kit; Human TNF-alpha Quantikine ELISA Kit, R&D Systems, Bio-Techne Corporation, Minneapolis, MN, USA).

#### Vascular function

Vascular function (main study outcome), including endothelial function and microvascular function, was evaluated according to guidelines [31, 32]. Briefly, the participant was laying in the supine position with their right arm extended while the brachial artery was imaged with ultrasound (8L-RS 4.0–13.0MHz probe, Vivid q, GE Healthcare, Mississauga, ON). Following one minute of baseline diameter imaging and blood flow data collection, a cuff was placed around the forearm distal to the ultrasound measuring site and inflated to supra-systolic pressure for five minutes. Reactive hyperemia was created by a sudden release of the cuff, and *microvascular function* was immediately evaluated as the velocity-time-integral of the outer envelope of the flow profile of the first pulse-wave of reactive hyperemia and normalized for heart rate (VHR) [31] (EchoPAC PC software, version 110.x.x, GE Healthcare, Horten, Norway). The microvascular function is thus a measure of how well the microvasculature distally of the cuff dilates in response to the ischemic stimuli [31, 32]. *Endothelial function* was subsequently assessed as the percent flow-mediated dilation (FMD) of the brachial artery compared to baseline in response caused by the reactive hyperemia, and normalized for baseline diameter [33] (Medical Imaging Applications, LLC, Coralville, IA, USA). The FMD is an indicator of how well the endothelium is responding to the increase in shear stress perpendicular to the vessel wall. Reduced endothelial function and microvascular function are both associated with cardiovascular risk [33, 34].

#### Arterial stiffness

Central arterial stiffness was evaluated between the carotid and femoral arteries using automated applanation tonometry (Complior, Alam Medical, Saint Quentin Fallavier, France) [35]. The distance between measuring sites (Δd) was divided by the time difference between the upslope of the pulse-waves at each measuring site (Δt) and arterial stiffness was expressed as the pulse wave velocity (PWV) = Δd/Δt [35].

### Divergences from the early protocol

This study reports data from a large research initiative registered on ClinicalTrails.gov (ID#: NCT02630511) in 2015. The study has since evolved and the following updates to the research procedures were made: a) pulmonary inflammatory markers were to be evaluated in exhaled breath condensate (EBC). Initial testing determined that EBC did not contain detectable levels of analytes and EBC testing was thus discontinued. b) The initial protocol planned for testing at 15 minutes, 1h and 24h following each challenge, which was proven unfeasible from operational and participant time-commitment perspectives. As such, data were only collected at the 1h time-point; and c) the analysis of inflammatory markers was planned to be outsourced to an external laboratory; however, more cost-effective options within our institution were discovered and used (see Outcome measures–systemic inflammation).

### Statistical analysis

Baseline characteristics for controls and asthma were summarized as mean with standard deviation (SD) for continuous variables and proportions for categorical variables. Differences in baseline clinical characteristics, including lung function and results from the cardiopulmonary exercise test, were evaluated using the student’s t-test or chi-square (χ^2^) test for continuous and categorical variables, respectively. The peak reduction in FEV_1_ were compared between groups of people with and without asthma during the different challenges using a 2-way repeated-measures analysis of variance (ANOVA), and between those with positive challenges in a one-way ANOVA. The vascular and inflammatory post-challenge responses to each challenge (placebo/mannitol/methacholine) among all individuals with and without asthma (control/asthma) were evaluated using mixed effects linear regression models while adjusting for order (random effect), age of participant, and pre-challenge values [36]. The models had fixed effects for group (reference: control), challenge (reference: placebo) and group by challenge interactions. To further explore the systemic inflammatory and vascular impact of positive airway challenges regardless of asthma status, the results from positive mannitol and methacholine challenges, and corresponding placebo challenges, were grouped separately and compared using linear regression models while adjusting for order (random effect) and pre-challenge values for each outcome. For this second set of analyses, the age was not included as a covariate due to the smaller sample size. Sample size calculations were performed based on preliminary data. Data collected in 11 asthmatics indicated a mean reduction in microvascular function of 20±9 cm following mannitol as compared to 12±13 cm with placebo with a correlation of 0.8 between the two measures. Based on these data, 24 participants would be sufficient to detect a difference between groups, assuming 80% power and α = 0.05. The unit of analysis was the participant. All statistical analyses were performed in SPSS Statistical software version 26.0.0.0 IBM Corp™, Armonk, NY, USA, and Studio Team 2020, RStudio: Integrated Development for R. RStudio, PBC, Boston, MA, USA.

## Results

### Participant characteristics

**Fig 1:**
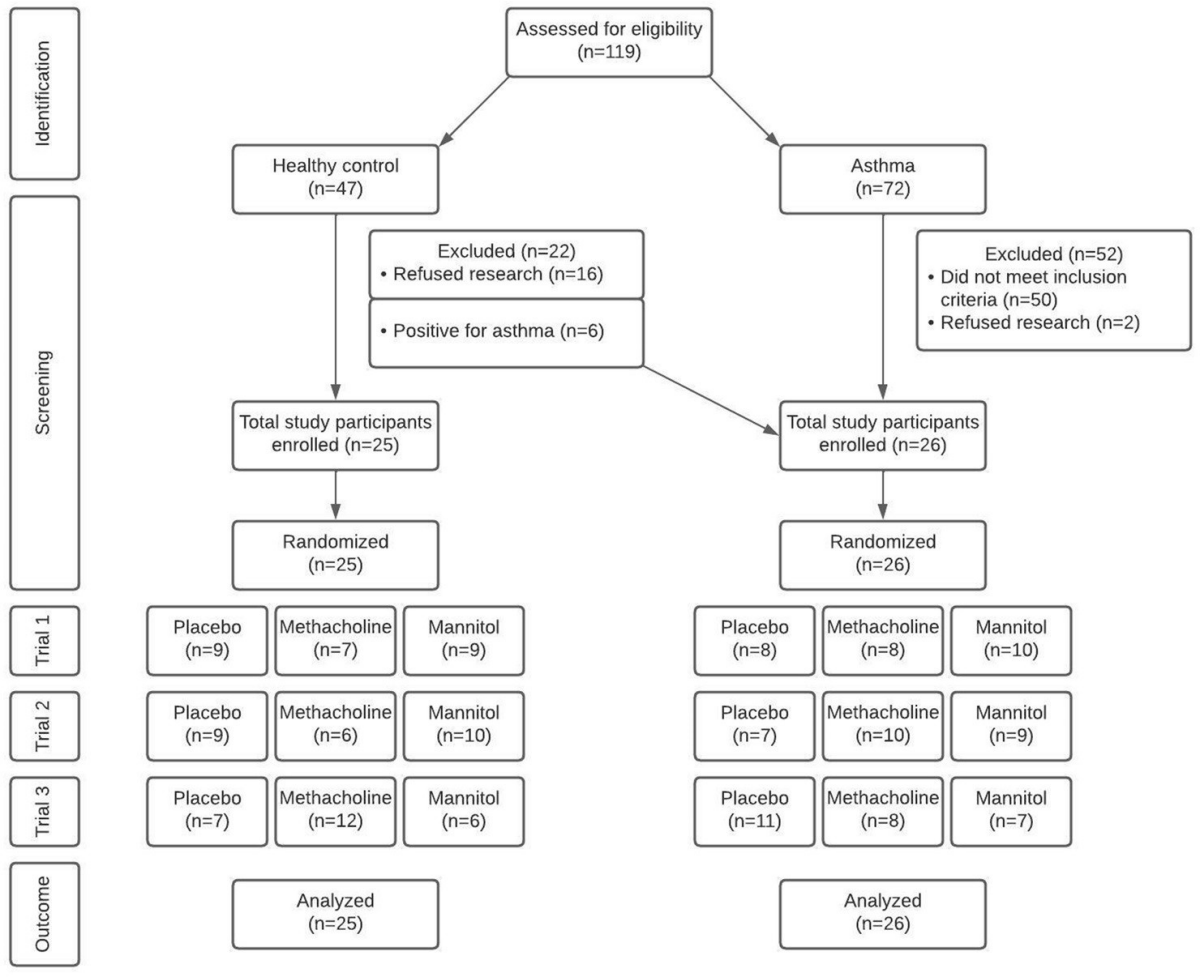
Consort diagram of recruitment, selection, testing, and analysis.

One hundred and nineteen potential participants were initially identified from the general population and through chart review. Of these, 25 control participants and 26 participants with asthma were included in the study (Fig 1). Baseline characteristics are outlined in Table 1. The groups were well matched for height and weight. A larger proportion of participants with asthma than controls had allergies (73 vs 16%, p<0.05). Pre bronchodilator FEV_1_ (86.6 vs 98.4% predicted, p<0.05) and FEV_1_/FVC (87.4 vs 96.8% predicted, p<0.05) were lower in asthma than controls.

**Table 1:** Clinical characteristics, control vs asthma. Values are expressed as mean (standard deviation) unless otherwise indicated. Lung function values reported were assessed without inhaled bronchodilators prior to testing.

	Controls	Asthma	p-value
Sample size, n (male/female)	25 (12/13)	26 (15/11)	0.488
Age, years	23.60 (3.81)	24.54 (5.15)	0.464
Height, m	1.73 (0.11)	1.73 (0.10)	0.992
Weight, kg	69.26 (11.29)	71.67 (12.19)	0.467
BMI, kg/m^2^	23.07 (1.79)	23.98 (3.18)	0.221
Allergies, n (% yes)	4 (16)	19 (73)	<0.001
ACQ score	0.04 (0.11)	0.66 (0.72)	<0.001
FEV_1,_ L	4.08 (0.89)	3.59 (0.81)	0.0469
FEV_1,_ % predicted	98.36 (12.72)	86.61 (12.73)	<0.005
FVC, L	4.98 (1.10)	4.91 (1.15)	0.819
FVC, % predicted	100.75 (10.74)	98.79 (11.22)	0.527
FEV_1_/FVC	81.98 (6.06)	73.51 (8.76)	<0.001
FEV_1_/FVC, % predicted	96.80 (7.71)	87.43 (10.48)	<0.001
TLC, L	6.39 (1.54)	6.43 (1.58)	0.935
TLC, % predicted	96.62 (11.90)	96.30 (11.03)	0.919
RV, L	1.33 (0.55)	1.46 (0.63)	0.439
RV, % predicted	87.86 (32.34)	95.64 (38.52)	0.440
DLCO, ml/min/mmHg	29.88 (7.85)	30.63 (7.72)	0.735
DLCO, % predicted	86.80 (9.46)	88.57 (11.03)	0.542

BMI: body mass index; ACQ: asthma control questionnaire; FEV_1_: forced expiratory flow in one second; FVC: forced vital capacity; TLC: total lung capacity; RV: residual volume; DLCO: diffusion capacity.

### Pulmonary responses to bronchial challenges

**Fig 2:**
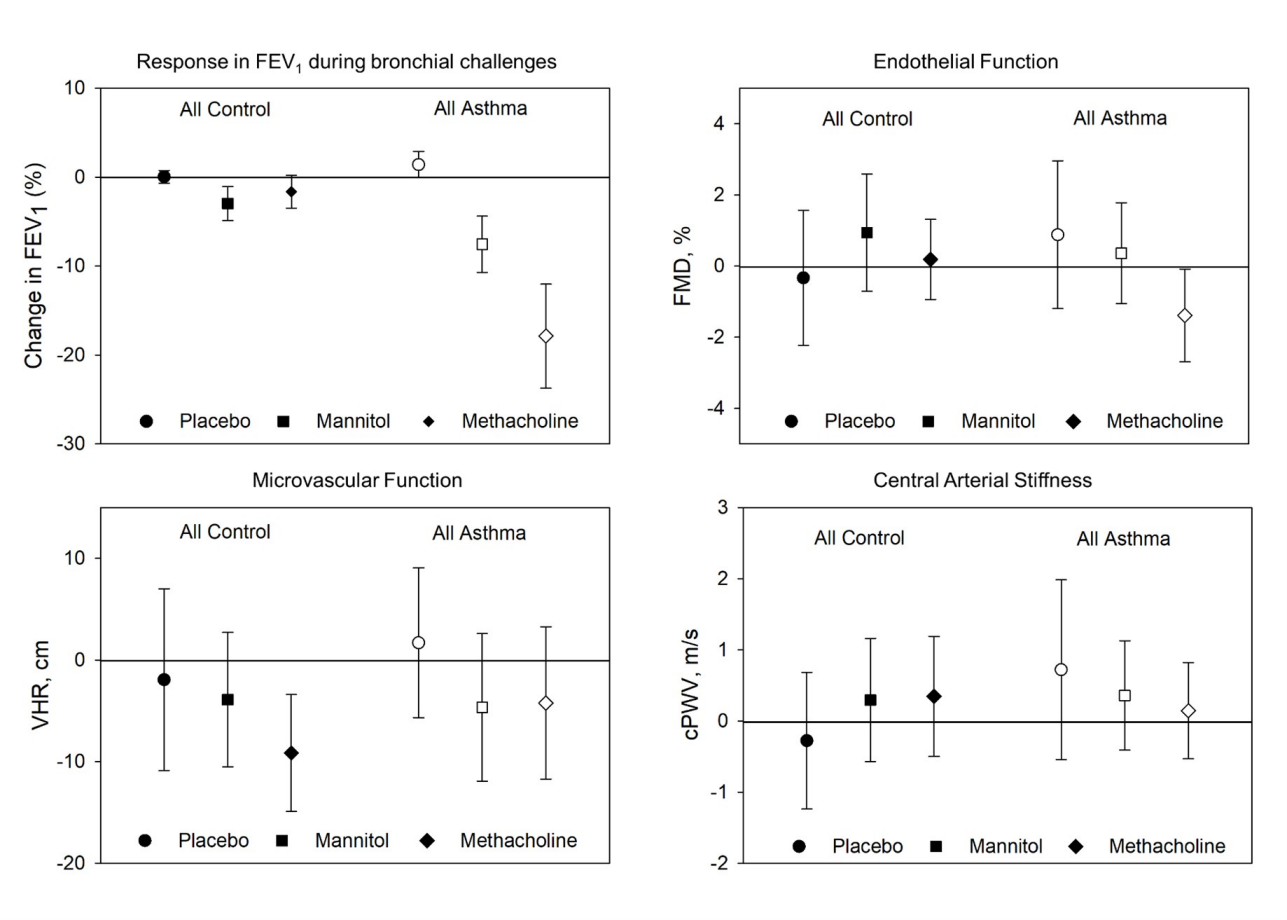
Unadjusted mean change and 95% Cl in FEV_1_ and vascular parameters (means and 95% Cl) among all participants regardless of responsiveness to challenges, grouped as control (filled markers) and asthma (open markers), challenges are placebo (circles), mannitol (squares), methacholine (diamonds). FMD: flow-mediated dilation; VHR: velocity time integral adjusted for heart rate; cPWV: central arterial stiffness.

The overall responses in FEV_1_ to placebo, mannitol, and methacholine (regardless of whether the tests were positive of negative) were 0.0% (95% Cl: -0.7 to 0.8%), -3.0% (95% Cl: -4.9 to -1.1%), and -1.6% (95% Cl: -3.5 to 0.2%) among controls and 1.4% (95% Cl: 0.0 to 2.9%), -7.5% (95% Cl: -10.7 to -4.4%), and -17.9% (95% Cl: -23.7 to -12.0%, p<0.05) in asthma, respectively (Fig 2A). In total, 14 people had a positive airway response to mannitol (FEV_1_: -14.2% [95% Cl: -17.0 to -11.4%]), and 13 people showed a positive airway response to methacholine (FEV_1_: -27.6% [95% Cl: -33.7 to -21.5%], p<0.05) (Fig 2B). Among the participants with positive mannitol and/or methacholine challenges (n = 20), the mean change in FEV_1_ during the placebo challenge was 1.0% (95% Cl: -0.7 to 2.6%) (Fig 2B).

### Systemic responses to bronchial challenges in all participants, grouped as asthma and control

Table 2 outlines the responses in the systemic inflammatory markers CRP, IL-6, and TNF measured immediately before and within the first hour following each bronchial challenge in all controls and people with asthma. The mixed effects linear regression models found markers of systemic inflammation unaffected by the type of challenge and group.

**Table 2:** Systemic inflammatory responses to bronchial challenges in controls vs asthma. Values are expressed as unadjusted means (SD).

	Placebo				Mannitol				Methacholine			
	Control		Asthma		Control		Asthma		Control		Asthma	
	Pre	Post	Pre	Post	Pre	Post	Pre	Post	Pre	Post	Pre	Post
CRP (mg/L)	1.19 (1.55)	1.00 (1.04)	1.60 (3.20)	0.79 (1.41)	0.96 (1.47)	0.91 (1.41)	1.65 (2.83)	1.13 (1.55)	0.96 (1.09)	1.26 (1.50)	0.57 (0.84)	0.99 (1.26)
TNF (pg/ml)	15.26 (33.25)	4.45 (10.98)	2.70 (6.37)	4.55 (15.58)	8.38 (15.61)	5.28 (10.89)	0.89 (3.25	3.25 (7.87)	3.05 (8.43)	3.45 (7.08)	2.88 (6.87)	4.77 (8.71)
IL-6 (pg/ml)	0.41 (1.91)	0.13 (0.31)	0.09 (0.31)	0.01 (0.06)	0.03 (0.10)	0.11 (0.37	0.51 (1.13)	0.24 (0.67)	0.08 (0.28)	0.03 (0.15)	0.00 (0.00)	0.00 (0.00)

CRP: C-reactive protein; TNF: tumor necrosis factor; IL-6: interleukin-6.

Fig 2 shows unadjusted mean vascular data for all challenges in all participants. The mixed effects linear regression models found both FMD and VHR were unaffected by type of challenge and group. PWV was increased in people with asthma compared to controls following the mannitol challenge as compared to placebo (estimated increase: 0.15 m/s, p = 0.05 for interaction); however no other interaction was observed.

### Systemic responses following positive bronchial challenges

**Fig 3:**
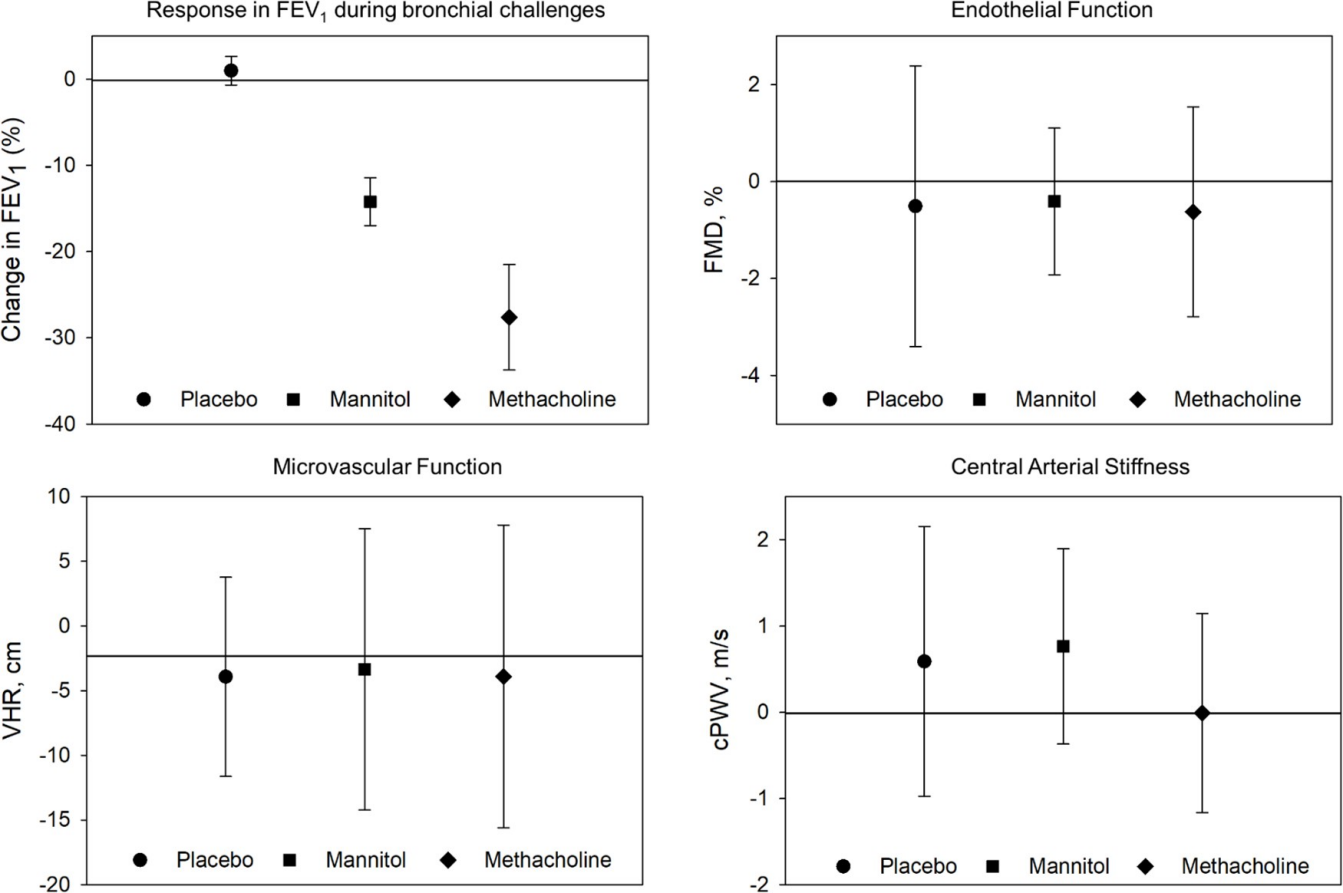
Unadjusted mean change and 95% Cl in FEV_1_ and vascular parameters following placebo, positive methacholine, and positive mannitol challenges. FMD: flow-mediated dilation; VHR: velocity time integral adjusted for heart rate; cPWV: central arterial stiffness.

The secondary analysis examined vascular and inflammatory responses among only those with a positive test (i.e., a reduction of ≥10% in FEV_1_ during the mannitol challenge or ≥20% reduction in FEV_1_ during the methacholine challenge) in comparison to the corresponding placebo challenges. The unadjusted mean values can be found in Fig 3 and Table 3. The linear regression analysis found that CRP, TNF and IL-6 were unaffected by the type of challenge. Similarly, FMD and VHR were unaffected by type of challenge indicating a positive challenge did not play a role in vascular function.

**Table 3:** Systemic inflammatory responses to positive airway challenges regardless of asthma status. Values are expressed as unadjusted means (SD).

	Placebo		Mannitol		Methacholine	
	Pre	Post	Pre	Post	Pre	Post
CRP (mg/L)	2.01 (2.15)	1.36 (1.61)	1.91 (1.88)	0.67 (1.40)	0.99 (0.73)	2.18 (1.71)
TNF (pg/ml)	3.42 (5.96)	2.69 (9.13)	8.00 (18.18)	5.93 (11.69)	2.87 (7.50)	6.20 (11.76)
IL-6 (pg/ml)	0.03 (0.12)	0.07 (0.21)	0.67 (1.40)	0.57 (0.89)	0.07 (0.20)	0.08 (0.23)

CRP: C-reactive protein; TNF: tumor necrosis factor; IL-6: interleukin-6.

## Discussion

We investigated the link between asthma and cardiovascular disease risk in a controlled laboratory environment by assessing the impact of the mannitol and methacholine airway challenges on systemic inflammation and vascular function in young people with confirmed asthma and in controls. Despite sufficiently reducing FEV_1_ through its suggested inflammatory pathway [18, 19, 21], mannitol did not elicit a clinically meaningful impact on systemic inflammation or vascular function as compared to the placebo challenge. Importantly, this study also showed that despite resulting in substantial bronchoconstriction (mean reduction in FEV_1_ of -27.6%), a positive methacholine challenge also failed to produce a measurable response in vascular function. As such, the results from this study questions whether the associated airway inflammation and bronchoconstriction immediately following an asthma exacerbation are sufficient to acutely impair vascular function and increase CV risk.

Vascular markers of cardiovascular disease risk are typically impaired in asthma compared to controls [5, 6], and asthma severity is correlated with the magnitude of cardiovascular risk [4–7, 10]. Most previous studies investigating cardiovascular risk in asthma have been observational. In this study, we attempted to provide the physiological link between asthma and elevated cardiovascular risk [2–4], and to confirm experimental work demonstrating that pulmonary inflammation leads to systemic inflammation conducted in animal models [14, 15] in a clinical laboratory setting. Previous work observed small but statistically significant changes in systemic levels of CRP and IL-6 when exposing otherwise healthy people to a pro-inflammatory insult, which resulted in both elevated arterial stiffness [17] and impairments in endothelial function [16]. Increases in CRP as large as 300% have been reported during a naturally occurring asthma exacerbation (compared to remission) [8]. However, while the mannitol airway challenge resulted in a mean peak reduction in FEV_1_ of -7.5% in all asthmatics (Fig 2), and -14.2% for positive mannitol challenges (Fig 3), the systemic inflammatory responses within the first hour following the airway challenge was not sufficient to result in meaningful changes in vascular function. Greater arterial stiffness was observed following the mannitol challenge in people with asthma but at an estimated increase in PWV of 0.15 m/s, this increase is well below any clinical importance [37]. To our knowledge, this is the first study to evaluate the systemic inflammatory and vascular responses to acute reductions in lung function. A naturally occurring asthma exacerbation may result in greater and more sustained inflammatory response and therefore more research on the influence of naturally occurring asthma exacerbations on vascular health is needed to fully understand the impact of pulmonary inflammation on cardiovascular risk in active asthma.

To normalize lung function after each challenge, and to minimize any discomfort from bronchoconstriction, each participant received 400°g salbutamol following each challenge irrespective of individual level of airway response. While salbutamol does not appear to have anti-inflammatory properties, salbutamol increases sympathetic activity [38], which would modulate vascular tone. The current study was carefully designed to examine the vascular responses to each challenge while at the same time adjusting for the corresponding pre-values [36] and further assessment of differences pre-post challenges would thus not have been appropriate. However, there is increased cardiovascular risk associated with inhaled beta-agonist use [39, 40] and we have previously shown that salbutamol acutely impairs vascular function in people with asthma who use short-acting beta-agonists regularly [41]. These findings suggest that the cardiovascular effects of inhaled salbutamol may play an important role in the increased cardiovascular risk observed with an asthma exacerbation.

The risk of developing cardiovascular disease increases with age [42] and it is possible that older asthmatics would have lower baseline vascular function and potentially a larger inflammatory and vascular response to airway inflammation. To avoid potential age-related bias in the data, participant age was adjusted for within the statistical model. Similarly, greater systemic responses may have been observed following airway inflammation among people with asthma with pre-existing cardiovascular disease. In the current study, young asthmatics with no cardiovascular disease were purposely recruited to allow for safe and careful examination of the link between airway inflammation, systemic inflammation, and vascular function, which may have limited the magnitude of responses seen in the outcomes. When possible to conduct safely, further research should focus on evaluating the impact of asthma exacerbations on systemic vascular health among elderly asthmatics with and without pre-existing cardiovascular disease.

A limitation of this study was the lack of confirmation of the pulmonary inflammatory responses to mannitol. While the EBC analysis did not yield detectable airway inflammation, other techniques such as induced sputum or bronchoscopy sampling would have likely confounded the vascular assessments. The mannitol inhalation challenge has previously been shown to increase markers of mast cell activation [18, 19], and anti-inflammatory asthma treatment significantly reduces the responsiveness to mannitol among asthmatics [21]. The change in FEV_1_ was thus used as a surrogate marker for pulmonary inflammation. In addition, it is possible that larger changes in systemic inflammation and vascular function and stiffness would have been seen in people with asthma if the mannitol challenges had been continued to elicit a 15% reduction instead of a 10% reduction in FEV_1_. The 10% cut-off was used as initial pilot work showed participant discomfort with reductions in FEV_1_ following mannitol beyond 10% of baseline.

In the current study, all follow-up measurements were done within the first hour following each challenge. Further assessments to evaluate changes over time were not feasible as the participants would have had to remain fasting and sedentary. Previous studies evaluating the effect of increased systemic inflammation on vascular function and arterial stiffness both observed detectable changes in inflammatory levels eight hours after the inflammatory insult [16, 17], with an additional increase in CRP and arterial stiffness reported at 32 hours [17]. It is thus plausible that the serum levels of inflammatory markers in the current study may have continued to rise beyond what was seen immediately after each challenge and have a delayed impact on the vascular function and arterial stiffness. Although immediate change was not noticed in the current study, further work to understand the impact of ongoing symptoms of asthma exacerbation and the relationship to inflammation and vascular function are needed.

## Conclusion

Airway challenges that induce bronchoconstriction (methacholine) or inflammation and bronchoconstriction (mannitol) did not result in immediate measurable or clinically meaningful acute changes in systemic inflammation or vascular function. These findings suggest that airway inflammation and bronchoconstriction immediately following asthma exacerbations are unlikely to be the sole mechanisms for the increased cardiovascular risk and other factors such as the impact of inhaler medications on cardiovascular health and the real-world impacts of asthma exacerbations need to be further examined.

## Supporting information

S1 Checklist CONSORT 2010 checklist of information to include when reporting a randomised trial*. (DOC)

S1 File Study dataset with anonymized data necessary to replicate study findings. (XLSX)

S2 File Study protocol. (DOCX)
