# Supplementary material for: Impact of airway challenges on cardiovascular risk in asthma – a randomized controlled trial
Source: PLoS One. 2023 Jul 17;18(7):e0288623. doi: 10.1371/journal.pone.0288623 (PMC10351735; doi:10.1371/journal.pone.0288623)
Supplement: S2 File — (DOCX) [file pone.0288623.s003.docx]

***The Impact of Asthma Exacerbations on Vascular Function***

| **Authors:** | *Dr. Michael Stickland, PhD^1^; Linn Moore, PhD^2^.* |
| --- | --- |
|  |  |
| **Institutions:** | *^1^Department of Medicine, Faculty of Medicine and Dentistry ^2^Faculty of Physical Education and Recreation* |
| **Corresponding Author:** | *Dr. Michael Stickland, PhD*  *Department of Medicine*  *3-135 Clinical Sciences Building*  *University of Alberta*  *Edmonton, Alberta*  *Canada*  *T6G 2J3*  *Ph. 780-492-3995*  *Fax. 780-492-4483*  *Email: michael.stickland@ualberta.ca* |

| Protocol/version #: Pro00047054  Current Version Date: 06/07/2023 |  |
| --- | --- |
| Previous IRB Approved Version Dates: n/a |  |

**List of Abbreviations**

ANOVA Analysis Of Variance

ACQ Asthma Control Questionnaire

CRP C - reactive protein

CV Cardiovascular

EBC Exhaled Breath Condensate

FEV_1_ Forced Expiratory Volume in the first second

FMD Flow-mediated dilation (% baseline)

FVC Forced Vital Capacity

HR Heart Rate

HREB Health Research Ethics Board

IL-6 Interleukin 6

IL-8 Interleukin 8

LT Lactate Threshold

PC_20_ Provocative Concentration yielding a 20% reduction in FEV_1_

PD_15_ Provocative dose yielding a 15% reduction in FEV_1_

PWV Pulse wave velocity (m/s)

RER Respiratory Exchange Ratio

SSRH Shear Stress during Reactive Hyperemia

TNFα Tumor Necrosis Factor alpha

VO_2max_ Maximal rate of oxygen consumption

VO_2peak_ Peak rate of oxygen consumption

VRH Velocity during Reactive Hyperemia

VTI Velocity Time Integral

**Table of Contents**

1 Introduction/Significance 6

2 Study Objectives 6

3 Patients and Methods 7

3.1 Study Design 7

3.1.1 General Design 7

3.1.2 Primary Outcome Variable 7

3.1.3 Secondary Outcome Variables 8

3.2 Subject Selection and Withdrawal 8

3.2.1 Inclusion Criteria 8

3.2.2 Exclusion Criteria 8

3.3 Study Procedures 8

3.4 Statistical Plan 11

3.4.1 Sample Size Determination 11

3.4.2 Statistical Methods 11

4 Data Handling and Record Keeping 11

4.1 Confidentiality 13

4.2 Records Retention 13

4.3 Regulatory Binder 14

*5* Study Auditing and Inspecting 14

6 Budget 14

7 Publication Plan 14

8 References 14

9 Attachments 17

# Introduction/Significance

This document is a clinical research protocol and the described study will be conducted in compliance with the protocol, Good Clinical Practices standards and associated Federal regulations (i.e Health Canada), and all applicable institutional research requirements.

Asthma is a chronic inflammatory disease characterized by pulmonary inflammation and bronchoconstriction, and the prevalence of asthma is steadily rising worldwide[^1^](#_ENREF_1). **Asthma and Cardiovascular Disease:**  While asthma is generally considered to be a disease of the airways, there are important systemic consequences which have predisposed people with asthma to become more likely to die from cardiovascular (CV) disease compared to non-asthmatics[^2^](#_ENREF_2). Additional CV risks have been reported in people with severe asthma[^3^](#_ENREF_3), and there is a relationship between reductions in lung function and cardiac death[^4^](#_ENREF_4). To date, little is known in regards to the interaction between asthma exacerbations and CV risk. **Asthma and vascular dysfunction:** Brachial flow-mediated dilation (FMD) is used as a non-invasive tool to evaluate endothelial function[^5^](#_ENREF_5). Brachial FMD is impaired in people with coronary dysfunction[^6^](#_ENREF_6), and can predict future CV events better than traditional CV risk factors[^7^](#_ENREF_7). People with asthma have previously been shown to have impaired endothelial function compared to non-asthmatics[^8^](#_ENREF_8), but the underlying mechanism(s) are unclear. **Asthma, Systemic Inflammation, and CV risk:** Chronic systemic inflammation is an established risk factor and predictor of future CV events[^9^](#_ENREF_9), and levels of systemic inflammation are increased in asthma[^10^](#_ENREF_10)^,^[^11^](#_ENREF_11). Furthermore, while people with high levels of inflammation are more likely to suffer both first time and recurrent cardiac events[^12^](#_ENREF_12)^,^[^13^](#_ENREF_13), levels of systemic inflammation in asthma are related to disease severity and are the highest during asthma exacerbations[^10^](#_ENREF_10)^,^[^11^](#_ENREF_11). Previous studies have shown that systemic inflammation directly impairs vascular function[^14^](#_ENREF_14), and rodent work has shown that pulmonary inflammation can impair vascular function[^15^](#_ENREF_15). To-date no study has examined how an asthma attack may affect vascular function and CV risk. Thus, to gain better understanding of the increased CV risks associated with asthma exacerbations, *the first aim of this study is to evaluate how acutely increasing pulmonary inflammation affects vascular function* in people with asthma. **Physical activity and the immune system:** Physical inactivity has previously been associated with increased systemic inflammation[^16^](#_ENREF_16), while higher levels of physical activity can reduce inflammation[^17^](#_ENREF_17) and vascular dysfunction[^18^](#_ENREF_18)^,^[^19^](#_ENREF_19). Acute exercise has been shown to modulate the systemic responses to inflammatory insults[^20^](#_ENREF_20), and being more physically active is associated with better asthma symptoms[^21^](#_ENREF_21) but whether acute exercise influences the systemic responses to asthma exacerbations is unknown. The second aim of this study is to *assess the influence of acute exercise on the systemic and vascular responses to acute pulmonary inflammation* in asthma.

# Study Objectives

***Primary Objective***

To examine the acute impact of pulmonary inflammation and bronchoconstriction on systemic inflammation and vascular function in asthma.

***Secondary Objective***

To evaluate the influence of *acute* exercise on systemic inflammatory and vascular responses to acute pulmonary inflammation.

# Patients and Methods

## Study Design

### General Design

This study is a case-control study utilizing a repeated measure design. Each participant will undergo a series of bronchial challenges trials, including

- Mannitol challenge
- Methacholine challenge
- Placebo (control) challenge.

Vascular parameters such as flow mediated dilation (FMD; endothelial function) and pulse wave velocity (PWV; arterial stiffness) will be evaluated before, immediately after, 1 hour after, and 24 hours after each challenge. Blood samples will be obtained for analysis of systemic inflammatory response and exhaled breath condensate will be collected for analysis of pulmonary inflammation and oxidative stress at each time-point.

For the secondary objective, the subjects will in a random order either exercise for 30 minutes or rest for 30 minutes before being subjected to either a placebo challenge or a mannitol challenge, in a cross-over design. The same parameters as for the primary objective will be evaluated before, immediately after, 1 hour after, and 24 hours after each challenge.

1. ***Case-control studies***

*Definition of study groups:*

Asthmatics, defined according to CTS criteria[^22^](#_ENREF_22), will be recruited for the study. This repeated measure design will minimize the risk of between-groups variability and match the case and the control group on all variables except the experimental parameters within the different trails.

*Blinding and avoiding bias:*

All subjects will be blinded to what bronchial challenge is administered. The challenges will be administered in random order and the research staff performing the vascular function analysis will be blinded to what challenge the participant has undergone within each trail.

### Primary Outcome Variable

Flow-mediated dilation (FMD) of the brachial artery following 5 minutes of forearm occlusion will be measured using our ultrasound machine (8L-RS 4.0-13.0 MHz probe, Vivid q, GE Healthcare, Mississauga, ON) and FMD data will be analyzed using FDA approved software available from Medical Imaging Applications (Coralville, IA, USA). FMD will be calculated as: (peak hyperemic diameter-baseline diameter)/baseline diameter x 100. Peak hyperemic brachial arterial velocity (and subsequently shear stress) will be determined using Doppler ultrasound, and used for normalization of FMD.

### Secondary Outcome Variables

Arterial stiffness will be determined using carotid – femoral pulse wave velocity (PWV), and PWV will be calculated from measurements of pulse transit time and the distance traveled by the pulse between recording sites.

## Subject Selection and Withdrawal

### Inclusion Criteria

To be eligible for this study, the participants have to be between the ages of 18 and 65, have a body mass index of less than 35 kg/m^2^, controlled or partly controlled asthma according to the Asthma Control Questionnaire[^23^](#_ENREF_23)^,^[^24^](#_ENREF_24).

### Exclusion Criteria

Subjects who at the time of the study have one or more risk factors for CV disease[^25^](#_ENREF_25), known CV disease, other lung diseases then asthma, current infections, or are pregnant will be excluded from the study.

## Study Procedures

*Measurements*

*Pulmonary function* – all participants will undergo a full pulmonary function test, according to established clinical guidelines[^26^](#_ENREF_26)^,^[^27^](#_ENREF_27). The expiratory volume in 1 second (FEV_1_) responses to 4x100 mg Salbutamol will be evaluated, and an increase in FEV_1_ of ≥ 12% and 200 ml will be considered positive for reversible airway constriction[^22^](#_ENREF_22).

*Endothelial function -* The endothelial function of the brachial artery will be evaluated after 10 minutes of rest in the supine position using ultrasound imaging (8L-RS 4.0-13.0MHz probe, Vivid q, GE Healthcare, Mississauga, ON). Baseline diameter of the brachial artery will be established, whereby the blood flow of the forearm will be occluded distally of the measuring site for the duration of 5 minutes. Upon release of the occlusion, the blood velocity and the brachial diameter will be monitored for 3 minutes, and later analyzed (Medical Imaging Applications, LLC, Coralville, IA, USA; EchoPAC PC software, version 110.x.x, GE Healthcare, Horten, Norway). Microvascular function will be evaluated as the velocity time integral-envelope of the first heartbeat of reactive hyperemia (VTI; m/s) and the velocity during reactive hyperemia (VRH) calculated as VTI x 60/HR[^28^](#_ENREF_28). Shear stress during reactive hyperemia (SSRH) will subsequently then evaluated as 8 x 0.035 x blood velocity/(baseline diameter/10) at peak velocity and as cumulative SSRH until the time of peak FMD%[^29^](#_ENREF_29)^,^[^30^](#_ENREF_30). FMD/SSHR will be considered the main outcome for endothelial function in this study, and VRH and SSRH as indicators of microvascular function. All tests will be conducted after a 12 hour fasting period. Where applicable, the subjects will be asked to withhold any long-acting asthma medication for a minimum on 48 hours prior to the test, and short-acting asthma medications for 12 hours prior to the test.

*Arterial stiffness* - Arterial stiffness will be evaluated using pulse wave velocity (PWV) between the carotid and femoral arteries. After 10 minutes of supine rest, the time of the up-stroke of 30 pulse waves will be collected simultaneously using applanation tonometry (Mikro-tip Catheter Transducers model SPT-301, MillarInstruments, Inc., Houston, TX, USA) over the femoral and the carotid arteries. The sites of measurement will be temporarily marked, whereby the distances between the markings and the sternal notch will be measured. The tonometry signal will be recorded using the Powerlab acquisition system (Powerlab, 16/30, ADInstruments, New South Wales, Australia). A total of 10 consecutive beats will be selected for analysis (LabChart version 7.3.5 ADInstruments, New South Wales, Australia) and the timing of each upstroke will be manually adjusted to minimize potential errors throughout the steps of analysis. After receiving a time-stamp for each beat, delta-time (Δt) will be calculated as the difference between the time of the up-stroke of the waveform at the femoral artery and the carotid artery (Δt = femoral waveform – carotid waveform). The distance (d) will be calculated as the difference between the distance from the sternal notch to the femoral artery and the sternal notch to the carotid artery. PWV was then calculated as d/Δt and expressed in meters per second (m/s)[^31^](#_ENREF_31). Beat-to-beat blood pressure will be measured throughout the test (Finapres Medical Systems, Amsterdam, The Netherlands) and the signal will be calibrated offline to brachial blood pressure also recorded at the time of testing. The heart rate will be monitored using 1-lead echocardiography (Dual Bio Amp, ADInstruments, New South Wales, Australia).

*Systemic inflammation -* To minimize number of venous punctuations, an intra-venous catheter will be inserted in an antecubital vein on the first day of each experimental day. Upon insertion, the catheter will be flushed with 0.9% NaCl. Blood will be collected before, immediately after, and 1 hour after after each challenge. Before each blood draw, approximately 3 ml of blood will be discarded whereby the sample will be collected. The catheter will then be flushed again with 0.9% NaCl. The catheter will be removed before the subject leaves the laboratory. A single blood draw will be performed at the 24 hour follow-up assessments. All blood samples will be collected in anti-coagulant-free tubes pre-labeled with the assigned subject identification number, date, and time-point. Once collected, the blood will be left allowed to clot in room temperature for a minimum of 30 minutes before being centrifuged at 1000g for 10 minutes at 4 degree Celsius. Serum will then be separated into aliquots of 100 µl immediately following centrifugation. The aliquots will be stored at -80 degrees Celsius and later outsourced to Eve Technologies, Calgary, AB, for analysis of inflammatory markers (i.e. of IL-6, IL-8, TNFα, and CRP).

*Pulmonary inflammation -* Exhaled breath condensate (EBC) will be collected in quantities of 1-2 mL before, immediately after, 1 hour after, and 24 hours after each bronchial challenge using commercially available collection tubes (RTube™ Respiratory Research, Inc., Austin, TX, USA). The subjects will be asked to breathe normally into a mouthpiece with their nose occluded for 10 minutes. The mouthpiece will be connected to a collection chamber that is covered in a cooling sleeve which allows for the exhaled breath to condense. A one-way valve keeps the EBC in the upper part of the collection tube, from where it will be extracted into aliquots for storage in -80°C until further processing. All EBC sample will be outsourced to Eve Technologies, Calgary, AB, for analysis for CRP and 8-isoprostane.

*Bronchial challenges*

*Mannitol challenge -* The mannitol challenge will be conducted according to the protocol created and validated to the methacholine challenge by the inventor[^32^](#_ENREF_32); dry powder mannitol (Aridol®, Pharmaxis Inc., Exton, PA, USA) will be inhaled in concentrations of 0, 5, 10, 20, 40, 80, 160, 160 and 160 mg mannitol, for a total cumulative dose of 653 mg. Spirometry, as described previously, will be performed before the first inhalation to receive baseline lung function values, and at 1 minute following each of the doses. The value for FEV_1_ following the inhalation of 0 mg mannitol will be the reference value used for comparison for the remainder of the challenge. The challenge will be considered positive if a cumulative concentration of ≤ 635 mg mannitol yield a reduction in FEV_1_ of ≥ 15% compared to baseline (0 mg mannitol) FEV_1_ (PD_15_; provocative dose yielding a 15% reduction in FEV_1_) whereby the challenge will end. A cumulative dose of 635 mg will be used as the maximum dose for when/if a mannitol challenge does not resulted in a ≥15% reduction in FEV_1_ (negative tests).

As shown previously[^32^](#_ENREF_32), the FEV_1_ will in most people spontaneous recovery to within 95% of baseline values within 60 minutes following the last mannitol inhalation and reliever medication will not be required. Capillary oxygen saturation will be monitored throughout the challenge (Radical 7, Masimo, Neuchatel, Switzerland), including the recovery, and the FEV_1_ will be measured every 10 minutes following the last inhalation of mannitol until the FEV_1_ has returned to within 95% of baseline values. In case of an unexpected severe bronchoconstriction following the last inhalation of mannitol, the subject will be given 400 mg Salbutamol (Ventolin® inhaler)[^32^](#_ENREF_32) to relax the airways. A physician will be available for supervision of all mannitol challenges.

*Methacholine challenge –* The participants will perform a baseline spirometry test whereby they will be asked to inhale cumulative concentrations of methacholine until there is ≥20% reduction in FEV_1_ or test last concentration (16 mg/ml) has been used. During the challenge, methacholine powder, diluted in sterile saline at concentrations of 0.031, 0.0625, 0.125, 0.25, 0.5, 1, 2, 4, 8, and 16 mg/ml, will be administered with a nebulizer according to a 2-minute tidal breathing protocol where the subject performs a FEV_1_ test 30 and 90 seconds after each 2 minutes of methacholine at incremental concentrations[^33^](#_ENREF_33). The tests will end if PC_20_ is reached or if the highest concentration of methacholine (16 mg/ml) is given[^22^](#_ENREF_22). If the test results in a reduction in FEV_1_ below 95% of baseline values, the FEV_1_ will be monitored every 10 minutes until spontaneous recovery to within 5% of baseline FEV_1_, or reversed using 4 puffs (100 mg/puff) Salbutamol. Capillary oxygen saturation will be monitored throughout the challenge (Radical 7, Masimo, Neuchatel, Switzerland).

*Placebo challenge -* The protocol for the placebo challenge will be identical to the protocol for the methacholine challenge, with the exception that no methacholine will be added to the inhaled saline mixture. The subject will be blinded to the fact that no methacholine has been added and will be monitored as if a potential severe bronchoconstriction could occur, identical to the methacholine challenge.

*Exercise trial*

*Cardiopulmonary exercise test* - A graded cardiopulmonary exercise test will be performed to determine the rate of oxygen consumption (VO_2max_) at maximal exercise and the power output at which the subjects reach their anaerobic threshold (AT). The test will be performed on a cycle ergometer (Ergoselect 200, Ergoline, Bitz, Germany) at incremental stages (25 watts increase/2 minute until the AT is reached, and then in steps of 25 watts/1 minute until exhaustion). While performing the fitness test, the subject will be breathing though a mouthpiece connected to a Vmax Metabolic Cart (CareFusion, Yorba Linda, CA, USA), allowing for breath to breath analysis of pulmonary gases. Throughout the test, the heart rate (HR) responses will be monitored, and ECG recorded. The subjects will also be asked to rate the level of perceived exertion at two-minute intervals during the test, expressed on a Borg scale from 1 (no exertion) to 10 (exhaustion). Blood pressure measurements will be taken at rest and every two minutes. VO_2max_ will be confirmed if the following criteria are met: 1) plateau in VO_2_, 2) respiratory exchange ratio (RER) ≥ 1.1, 3) HR ≥ 90 % of predicted maximum, 4) patient exhaustion/Borg scale > 9/10, or 5) evidence of respiratory limitations. In the absence of a plateau in VO_2_ where remaining criteria for a maximal test were met, the highest recorded VO2 value will be referred to as VO_2peak_ and used as a substitute for VO_2max_ as a measurement of cardio-pulmonary fitness[^34^](#_ENREF_34).

The power output at AT will be noted, and a power output of 25 watts below the AT power output will be chosen for the 30 minute exercise protocol for the second objective of the study. In addition, the pulmonary responses to exercise will be established by spirometry before and after exercise.

## Statistical Plan

### Sample Size Determination

We do not know the anticipated effect of our interventions on FMD; however, based on previous studies[^8^](#_ENREF_8), a total of 20 subjects per group will be sufficient to detect a difference in FMD between trials (α=0.05, power=0.8). An additional 10 subjects per group will be recruited to account for dropouts. Thus, 60 subjects will be recruited for aim 1 and 60 subjects will be recruited for aim 2.

### Statistical Methods

For the primary aim, data will be summarized as means ± standard deviation. Baseline inflammatory and vascular values will be compared across trials using a one-way ANOVA followed by Tukey’s post-hoc test. A 2-way ANOVA will be used to evaluate potential interactions between Trial 1, 2, and 3, and time of measurement (baseline, immediately post-challenge, 1 hour post-challenge, and 1 day post-challenge). For the secondary aim, baseline values will be compared using a one-way ANOVA. Mean values in pulmonary inflammation, systemic inflammation, and vascular function between the exercise and the resting protocol, challenge or placebo, and time of measurement will be evaluated using a 3-way ANOVA.

# Data Handling and Record Keeping

***Data to be collected:***

**Outcome variables:**

Endothelial function

*Source of the data:* Flow-mediated dilation test (see *measurement, endothelial function* above).

*Time point for collection:* At baseline, immediately after each bronchial challenge, 1 hour after each challenge and at 24 hours after each challenge.

*Who will collect the data:* All data will be collected by trained research staff associated with the study.

*Why the data is being collected:* To evaluate the influence of acute pulmonary inflammation on vascular health, and the influence of exercise on vascular dysfunction following acute inflammation.

*Is the data from a standardized tool:* Yes.

*What form will the data will take (e.g. binary, continuous (numeric), time to event):* Continuous.

Arterial stiffness

*Source of the data:* pulse wave velocity test (see *measurements, arterial stiffness* above).

*Time point for collection:* At baseline, immediately after each bronchial challenge, 1 hour after each challenge, and at 24 hours after each challenge.

*Who will collect the data:* All data will be collected by trained research staff associated with the study.

*Why the data is being collected:* To evaluate the influence of acute pulmonary inflammation on vascular health, and the influence of exercise on vascular dysfunction following acute inflammation.

*Is the data from a standardized tool:* Yes.

*What form will the data will take (e.g. binary, continuous (numeric), time to event):* Continuous.

Systemic inflammation

*Source of the data:* Venous blood samples (see *measurements, systemic inflammation* above).

*Time point for collection:* At baseline, immediately after each bronchial challenge, 1 hour after each challenge, and at 24 hours after each challenge.

*Who will collect the data:* All data will be collected by trained research staff associated with the study.

*Why the data is being collected:* To evaluate changes in systemic inflammatory levels throughout the study protocol.

*Is the data from a standardized tool:* Yes.

*What form will the data will take (e.g. binary, continuous (numeric), time to event):* Continuous.

Pulmonary inflammation

*Source of the data:* Exhaled breath condensate (see *measurements, pulmonary inflammation* above).

*Time point for collection:* At baseline, immediately after each bronchial challenge, 1 hour after each challenge, and at 24 hours after each challenge.

*Who will collect the data:* All data will be collected by trained research staff associated with the study.

*Why the data is being collected:* To evaluate changes in pulmonary inflammatory levels throughout the study protocol.

*Is the data from a standardized tool:* Yes.

*What form will the data will take (e.g. binary, continuous (numeric), time to event):* Continuous.

**Explanatory variables and potential cofounding variables:**

Subject demographics

*Source of the data:* interview with subject.

*Time point for collection:* during a pre-test session.

*Who will collect the data:* All data will be collected by trained research staff associated with the study.

*Why the data is being collected:* To ensure subject meet the inclusion/exclusion requirements, and to obtain general sample characteristics.

*Is the data from a standardized tool:* No, subject demographics will be collected according to

*What form will the data will take (e.g. binary, continuous (numeric), time to event):* Nominal or ordinal.

Perceived level of control

*Source of the data:* The asthma control questionnaire .

*Time point for collection:* during a pre-test session, and at each baseline time-point.

*Who will collect the data:* All data will be collected by trained research staff associated with the study.

*Why the data is being collected:* To evaluate the current levels of perceived asthma control at each study day.

*Is the data from a standardized tool:* Yes.

*What form will the data will take (e.g. binary, continuous (numeric), time to event):* Continuous.

Lung function

*Source of the data:* Pulmonary function test (see *measurements, pulmonary function* above)

*Time point for collection:* during a pre-test session, and at each baseline time-point.

*Who will collect the data:* All data will be collected by trained research staff associated with the study.

*Why the data is being collected:* To evaluate baseline lung function at each study day.

*Is the data from a standardized tool:* Yes.

*What form will the data will take (e.g. binary, continuous (numeric), time to event):* Continuous.

Data collection forms and questionnaires are included as appendices.

## Confidentiality

Each research participant will be assigned a study ID which will not be associated with any personal identifiable information. All collected study data will be stored either on password protected computer hard drives or in locked filing cabinets.

## Records Retention

All data will be stored for 5 years following the completion of the study, whereby it will be destroyed.

## Regulatory Binder

The investigator will maintain a regulatory binder containing all information pertinent to the study.

# Study Auditing and Inspecting

Not applicable.

# Budget

Source of Funding: This study will be funded by Dr. Stickland’s operating grant.

For complete budget, please see appendix 1.

Budget summary:

| Salaries and Benefits | | $14,985 |
| --- | --- | --- |
| Materials and Supplies | | $23,842 |
| Payment to Subjects | | $4,096 |
| Publications | | $2,000 |
| **TOTAL BUDGET** | **$44,923** | |
|  |  | |

# Publication Plan

Upon completion, this study is expected to be published as two separate peer-reviewed research articles.

# References

***1.*** *TheAsthmaSocietyOfCanada. Asthma Facts & Statistics (*[*http://www.asthma.ca/corp/newsroom/pdf/asthmastats.pdf)*](http://www.asthma.ca/corp/newsroom/pdf/asthmastats.pdf))*.*

***2.*** *Iribarren C, Tolstykh IV, Miller MK, Sobel E, Eisner MD. Adult asthma and risk of coronary heart disease, cerebrovascular disease, and heart failure: a prospective study of 2 matched cohorts. Am J Epidemiol. Dec 1 2012;176(11):1014-1024.*

***3.*** *Toren K, Lindholm NB. Do patients with severe asthma run an increased risk from ischaemic heart disease? Int J Epidemiol. Jun 1996;25(3):617-620.*

***4.*** *Sin DD, Wu L, Man SF. The relationship between reduced lung function and cardiovascular mortality: a population-based study and a systematic review of the literature. Chest. Jun 2005;127(6):1952-1959.*

***5.*** *Corretti MC, Anderson TJ, Benjamin EJ, et al. Guidelines for the ultrasound assessment of endothelial-dependent flow-mediated vasodilation of the brachial artery: a report of the International Brachial Artery Reactivity Task Force. J Am Coll Cardiol. Jan 16 2002;39(2):257-265.*

***6.*** *Anderson TJ, Uehata A, Gerhard MD, et al. Close relation of endothelial function in the human coronary and peripheral circulations. J Am Coll Cardiol. Nov 1 1995;26(5):1235-1241.*

***7.*** *Lau KK, Chan YH, Yiu KH, et al. Incremental predictive value of vascular assessments combined with the Framingham Risk Score for prediction of coronary events in subjects of low-intermediate risk. Postgrad Med J. Mar 2008;84(989):153-157.*

***8.*** *Yildiz P, Oflaz H, Cine N, et al. Endothelial dysfunction in patients with asthma: the role of polymorphisms of ACE and endothelial NOS genes. J Asthma. Apr 2004;41(2):159-166.*

***9.*** *Cesari M, Penninx BW, Newman AB, et al. Inflammatory markers and onset of cardiovascular events: results from the Health ABC study. Circulation. Nov 11 2003;108(19):2317-2322.*

***10.*** *Yokoyama A, Kohno N, Fujino S, et al. Circulating interleukin-6 levels in patients with bronchial asthma. Am J Respir Crit Care Med. May 1995;151(5):1354-1358.*

***11.*** *Zietkowski Z, Tomasiak-Lozowska MM, Skiepko R, Mroczko B, Szmitkowski M, Bodzenta-Lukaszyk A. High-sensitivity C-reactive protein in the exhaled breath condensate and serum in stable and unstable asthma. Respir Med. Mar 2009;103(3):379-385.*

***12.*** *Ridker PM, Rifai N, Pfeffer M, Sacks F, Lepage S, Braunwald E. Elevation of tumor necrosis factor-alpha and increased risk of recurrent coronary events after myocardial infarction. Circulation. May 9 2000;101(18):2149-2153.*

***13.*** *Ridker PM, Rifai N, Stampfer MJ, Hennekens CH. Plasma concentration of interleukin-6 and the risk of future myocardial infarction among apparently healthy men. Circulation. Apr 18 2000;101(15):1767-1772.*

***14.*** *Hingorani AD, Cross J, Kharbanda RK, et al. Acute systemic inflammation impairs endothelium-dependent dilatation in humans. Circulation. Aug 29 2000;102(9):994-999.*

***15.*** *Hazarika S, Van Scott MR, Lust RM, Wingard CJ. Pulmonary allergic reactions impair systemic vascular relaxation in ragweed sensitive mice. Vascul Pharmacol. Nov-Dec 2010;53(5-6):258-263.*

***16.*** *Hamer M, Sabia S, Batty GD, et al. Physical activity and inflammatory markers over 10 years: follow-up in men and women from the Whitehall II cohort study. Circulation. Aug 21 2012;126(8):928-933.*

***17.*** *Milani RV, Lavie CJ, Mehra MR. Reduction in C-reactive protein through cardiac rehabilitation and exercise training. J Am Coll Cardiol. Mar 17 2004;43(6):1056-1061.*

***18.*** *DeSouza CA, Shapiro LF, Clevenger CM, et al. Regular aerobic exercise prevents and restores age-related declines in endothelium-dependent vasodilation in healthy men. Circulation. Sep 19 2000;102(12):1351-1357.*

***19.*** *Dod HS, Bhardwaj R, Sajja V, et al. Effect of intensive lifestyle changes on endothelial function and on inflammatory markers of atherosclerosis. Am J Cardiol. Feb 1 2010;105(3):362-367.*

***20.*** *Starkie R, Ostrowski SR, Jauffred S, Febbraio M, Pedersen BK. Exercise and IL-6 infusion inhibit endotoxin-induced TNF-alpha production in humans. FASEB J. May 2003;17(8):884-886.*

***21.*** *Mancuso CA, Choi TN, Westermann H, Wenderoth S, Wells MT, Charlson ME. Improvement in asthma quality of life in patients enrolled in a prospective study to increase lifestyle physical activity. J Asthma. Feb 2013;50(1):103-107.*

***22.*** *Lougheed MD, Leniere C, Ducharme FM, et al. Canadian Thoracic Society 2012 guideline update: Diagnosis and management of asthma in preschoolers, children and adults: executive summary. Can Respir J. Nov-Dec 2012;19(6):e81-88.*

***23.*** *Juniper EF, Bousquet J, Abetz L, Bateman ED. Identifying 'well-controlled' and 'not well-controlled' asthma using the Asthma Control Questionnaire. Respir Med. Apr 2006;100(4):616-621.*

***24.*** *Juniper EF, O'Byrne PM, Guyatt GH, Ferrie PJ, King DR. Development and validation of a questionnaire to measure asthma control. Eur Respir J. Oct 1999;14(4):902-907.*

***25.*** *Pescatello LS. American College of Sport Medicine's Guidelines for Exercise Testing and Perscription.*

***26.*** *Miller MR, Hankinson J, Brusasco V, et al. Standardisation of spirometry. Eur Respir J. Aug 2005;26(2):319-338.*

***27.*** *Pellegrino R, Viegi G, Brusasco V, et al. Interpretative strategies for lung function tests. Eur Respir J. Nov 2005;26(5):948-968.*

***28.*** *Philpott AC, Lonn E, Title LM, et al. Comparison of new measures of vascular function to flow mediated dilatation as a measure of cardiovascular risk factors. Am J Cardiol. Jun 1 2009;103(11):1610-1615.*

***29.*** *Harris RA, Nishiyama SK, Wray DW, Richardson RS. Ultrasound assessment of flow-mediated dilation. Hypertension. May 2010;55(5):1075-1085.*

***30.*** *Thijssen DH, Black MA, Pyke KE, et al. Assessment of flow-mediated dilation in humans: a methodological and physiological guideline. Am J Physiol Heart Circ Physiol. Jan 2011;300(1):H2-12.*

***31.*** *Laurent S, Cockcroft J, Van Bortel L, et al. Expert consensus document on arterial stiffness: methodological issues and clinical applications. Eur Heart J. Nov 2006;27(21):2588-2605.*

***32.*** *Anderson SD, Brannan J, Spring J, et al. A new method for bronchial-provocation testing in asthmatic subjects using a dry powder of mannitol. Am J Respir Crit Care Med. Sep 1997;156(3 Pt 1):758-765.*

***33.*** *Crapo RO, Casaburi R, Coates AL, et al. Guidelines for methacholine and exercise challenge testing-1999. This official statement of the American Thoracic Society was adopted by the ATS Board of Directors, July 1999. Am J Respir Crit Care Med. Jan 2000;161(1):309-329.*

***34.*** *Stickland MK, Butcher SJ, Marciniuk DD, Bhutani M. Assessing exercise limitation using cardiopulmonary exercise testing. Pulm Med. 2012;2012:824091.*

# Attachments

Appendix 1. Budget

**1. Salaries and Benefits**

**Research Assistant**: Funds are requested for the salary one research assistant (0.25 full-time equivalents, FTE). This person will assist with data collection for all aims and ensure quality control of all data collection and data analysis.

| FTE | 1.0 | 0.25 |
| --- | --- | --- |
| Salary (Grade 6, Step 5): | $46,155 | $11,539 |
| Benefits (23%): | $13,786 | $3,447 |
| **Total Salaries and Benefits** |  | **$14,985** |

**2. Material and Supply**

**Asthma Challenges Kits**: Methacholine challenges will be performed on 16 subjects to evaluate the inflammatory and vascular responses to bronchoconstriction. Mannitol challenges will be evaluated on the same 16 subjects during rests and following acute exercise, i.e. a total of 32 mannitol challenges will be conducted.

| Aridol (Mannitol) 32 X $80.14 | $2,564 |
| --- | --- |
| Methacholine 16 X $50.00 | $800 |
| **Total Asthma Challenges Kits** | **$3,364** |

**Exhaled Breath Condensate (EBC)**: EBC will be collected using the RTube™ EBC collector before and 1 and 24 hour following each asthma challenge (4 challenges X 16 subjects X 3 time-points = 192 samples) for analysis of CRP (see attached quote from Eve technologies).

| RTube™ EBC 100 kit (2x) | $6,060 |
| --- | --- |
| EBC analysis (attached quote) CRP (n=192) | $3,720 |
| **Total EBC** | **$9,780** |

**Systemic Inflammatory Markers**: Venous blood will be collected and processed for serum analysis before and 1 and 24 hour following each asthma challenge (4 challenges X 16 subjects X 3 time-points = 192 samples) for analysis of IL-6, TNFα, CRP (see attached quote from Eve technologies). The anti-inflammatory cytokine IL-10 will be analyzed serum before and 1 and 24 hour following the exercise/mannitol challenge (1 challenge X 16 subjects X 3 time-points = 48 samples).

| Blood collection supplies (vacutainers, alcohol wipes, needles) | $500 |
| --- | --- |
| Blood analysis (see attached quote) |  |
| IL-6, TNFα, CRP (n=192 in duplicates (384)) and IL-10 (n=48 in duplicates (96)) | $9,698 |
| **Total Systemic Inflammatory Markers** | **$10,198** |
|  |  |

**Other Supplies**: A CPET will be performed on all participants to determine peak oxygen consumption. Respiratory gas exchange data will be obtained, with oxygen consumption, ventilation, heart rate, rating of perceived exertion and dyspnea recorded. All will perform the test with a 12-lead ECG. These funds are required as all these expendables are needed to do our basic experiments.

| Metabolic Measurement: mouthpieces, mouth filters, ECG electrodes, pulse oximetry electrodes, ultrasound jelly. | $500 |
| --- | --- |
| **Total Other Supplies** | **$500** |

| **Total Material and Supplies** | **$23,842** |
| --- | --- |

**3. Payment to Study Subjects**

**Parking and Honoraria**: All research participants will be reimbursed for parking at the University of Alberta East Parkade (4 challenges X 2 days X 16 participants = 128 visits). Participants who complete all parts of the study will receive a $200 gift card (on average - $25/visit) at their choice.

| Parking ($7/day) | $896 | |
| --- | --- | --- |
| Honoraria ($200/subject) | $3,200 | |
| **Total Payment to Subjects** | | **$4,096** |

**4. Publication Cost**

**Publications**: due to the different foci in Aim 1 and Aim 2, two separate papers will be written based on the results from this proposed study.

| Paper 1 - aim 1 | $1,000 |
| --- | --- |
| Paper 2 - aim 2 | $1,000 |
| **Total Publications** | **$2,000** |

**5. Summary**

| Salaries and Benefits | | $14,985 |
| --- | --- | --- |
| Materials and Supplies | | $23,842 |
| Payment to Subjects | | $4,096 |
| Publications | | $2,000 |
| **TOTAL BUDGET** | **$44,923** | |
